# Genomic characteristics of *Vibrio vulnificus* strains isolated from clinical and environmental sources

**DOI:** 10.1186/s44342-024-00029-w

**Published:** 2024-11-27

**Authors:** Jinkyeong Lee, Jeong-Ih Shin, Woo Young Cho, Kun Taek Park, Yeun-Jun Chung, Seung-Hyun Jung

**Affiliations:** 1 Department of Medical Sciences, Graduate School of The Catholic University of Korea, Seoul, 06591 Republic of Korea; 2 Precision Medicine Research Center, Catholic Research Institute for Human Genome Polymorphism, College of Medicine, The Catholic University of Korea, Seoul, 06591 Republic of Korea; 3 ConnectaGen, Hanam, 12918 Republic of Korea; 4 Department of Biological Sciences, Inje University, Gimhae, 50834 Republic of Korea; 5 Department of Microbiology, College of Medicine, The Catholic University of Korea, Seoul, 06591 Republic of Korea; 6 Department of Biochemistry, College of Medicine, The Catholic University of Korea, Seoul, 06591 Republic of Korea

**Keywords:** *Vibrio vulnificus*, Whole-genome sequencing, Antimicrobial resistance, Virulence factor, Multi-locus sequence typing

## Abstract

*Vibrio vulnificus*, a gram-negative pathogenic bacterium, transmitted via undercooked seafood or contaminated seawater, causes septicemia and wound infections. In this study, we analyzed 15 clinical and 11 environmental isolates. In total, 20 sequence types (STs), including eight novel STs, were identified. Antibiotic resistance gene analysis commonly detected the cyclic AMP receptor protein (CRP) in both the clinical and environmental isolates. Interestingly, clinical and environmental isolates were non-susceptible to third-generation cephalosporins, such as ceftazidime and cefotaxime, complicating the treatment of *V. vulnificus* infection. Multiple antibiotic resistance (MAR) index ranged from 0.1 to 0.5, with clinical isolates showing a higher mean MAR index than the environmental isolates, indicating their broader spectrum of resistance. Notable, no quantitative (124.3 vs. 126.5) and qualitative (adherence, antiphagocytosis, and chemotaxis/motility) differences in virulence factors were observed between the environmental and clinical strains. The molecular characteristics identified in this study provide insights into the virulence of *V. vulnificus* strains in South Korea, highlighting the need for continuous surveillance of antibiotic resistance in emerging *V. vulnificus* strains.

**Supplementary Information:**

The online version contains supplementary material available at 10.1186/s44342-024-00029-w.

## Introduction

*Vibrio vulnificus* is a gram-negative aquatic bacterium naturally found in warm coastal water, especially in high salinity regions [1]. It belongs to the Vibrionaceae family and is closely related to *Vibrio cholerae*, the causative agent of cholera. *V. vulnificus* is an opportunistic pathogen commonly transmitted through the consumption of seafood, particularly raw or undercooked shellfish, such as oysters [2]. In rare cases, it enters the body through the exposure of open wounds to contaminated water, leading to invasive infections [2]. *V. vulnificus* causes various infections, ranging from gastroenteritis to life-threatening septicemia, exhibiting the highest fatality rate among foodborne pathogens [3]. It only causes mild infections in healthy individuals but poses a significant risk to individuals with a weak immune system, particularly those with liver disease or diabetes [4]. In South Korea, 69 cases of *V. vulnificus* sepsis with 27 deaths and a fatality rate of 39.1% were reported in 2023 [5].

Virulence factors affect the infection capacity of *V. vulnificus*, playing important roles in its pathogenicity [6]. For example, *vvhA* gene related to *V. vulnificus* virulence is used by public health authorities to assess its human pathogenicity [7]. Moreover, *vcgC* and *vcgE* are useful markers to distinguish between its clinical and environmental strains [8]. *vcgC* is commonly detected in human-infecting strains, whereas *vcgE* is widely used to identify environmental strains [7, 9]. Specifically, *vcgC* produces proteins for host invasion, whereas *vcgE* produces proteins to respond to environmental stress and evade the host immune responses [7]. However, no definitive consensus has been reached on whether specific virulence factors distinguish between the pathogenic and nonpathogenic strains of *V. vulnificus* [10]. In addition to virulence, antibiotic resistance is another important factor associated with *V. vulnificus* infections [11, 12]. This resistance is transmitted via clonal transmission and horizontal gene transfer, further complicating infection control and treatment [13, 14]. Therefore, virulence profiles must be analyzed to assess the pathogenic potential and monitor the antibiotic resistance of pathogenic isolates.

In this study, we isolated 26 V*. vulnificus* strains from coastal water and infected patients and analyzed them via whole-genome sequencing (WGS) to (i) investigate their molecular subtypes and diversity and (ii) examine their genomic characteristics, including antimicrobial resistance genes and virulence factors.

## Materials and methods

### Bacterial collection and culture

In South Korea, 11 V*. vulnificus* strains were collected between August and September, 2022, for national foodborne pathogen surveillance research. *V. vulnificus* strains were isolated from the coastal water of five provinces in South Korea (three from Namhae-gun, Gyeongsangnam-do, two from Geoje-si, Gyeongsangnam-do, three from Mokpo-si, Jeollanam-do, two from Busan, and one from Ulsan). Additionally, 15 clinical *V. vulnificus* strains were isolated from nationwide bacterial biobanks in Korea (GNUH-NCCP) during 2010–2020. Of the 15 clinical strains, 11 were isolated from blood samples and 4 from pus samples. The details of each isolate are provided in Supplementary Table S1. *V. vulnificus* strains were cultured on blood agar plates at 37 °C under ambient air conditions for 18 to 24 h and stored at − 80 °C until analysis. This study was approved by the Institutional Review Board of the Catholic University of Korea College of Medicine (approval number: MC24SASI0019), and the requirement for informed consent was waived.

### Drug susceptibility testing

Antimicrobial susceptibility testing for 15 drugs (gentamicin [GEN], meropenem [MEM], chloramphenicol [CHL], cefoxitin [FOX], cefotaxime [CTX], ceftazidime [CAZ], cefepime [FEP], ampicillin [AMP], colistin [COL], tetracycline [TET], amoxicillin/clavulanic acid [AMC], nalidixic acid [NAL], ciprofloxacin [CIP], sulfisoxazole [FIS], and trimethoprim/sulfamethoxazole [SXT]) was performed with the Sensititre TM KRNV6F Kit (Thermo Fisher Scientific, Waltham, MA, USA) using the microbroth dilution method. Antimicrobial breakpoints were determined according to the Clinical and Laboratory Standards Institute guideline [15], with minimum inhibitory concentration (MIC) not in the “S” range indicating resistance. *Escherichia coli* ATCC 25922 was used as the standard strain. For antibiotics without established resistance determination criteria (COL, NAL, and FIS), MIC50 and MIC90 values were used to assess their susceptibility. Three strains (two environmental and one clinical) were not tested for drug susceptibility owing to their lack of regrowth.

Multiple antibiotics resistance (MAR) method described by Osundiya et al. [16] was used to determine the MAR index [17]. MAR index is calculated as the ratio of the number of antibiotics to which the organism is resistant divided by the total number of antibiotics to which the organism is exposed. MAR index ≥ 0.2 was defined as a high risk of the used antibiotics [13].

### Whole-genome sequencing analysis

Genomic DNA was extracted using the iDetect gDNA Prep Kit for Microbes (ConnectaGen, Hanam, South Korea) and evaluated using NanoDrop spectrophotometer (Thermo Fisher Scientific) and Qubit 2.0 Fluorometer (Thermo Fisher Scientific). Next-generation sequencing libraries were constructed using the TruSeq Nano DNA Sample Preparation Kit (Illumina, San Diego, CA, USA). The sequencing libraries were further pooled and sequenced using the Illumina NovaSeq 6000 System. Sequencing adapters and low-quality bases were trimmed using the Trimmomatic software. The trimmed reads were assembled using SPAdes [18], and the assembled contigs were annotated and evaluated using Prokka [19] and Quast [20], respectively.

Kraken2 was used to confirm the species of each strain [21]. Multi-locus sequence types (STs) were determined using the MLST tool (https://github.com/tseemann/mlst) with allelic profiles of 10 housekeeping genes (*glp*, *gyrB*, *mdh*, *metG*, *purM*, *dtdS*, *lysA*, *pntA*, *pyrC*, and *tnaA*) from the PubMLST database [22]. Newly discovered STs were submitted to the PubMLST database. Antimicrobial resistance genes and virulence factors were identified using ABRicate employing the Comprehensive Antibiotic Resistance Database [23] and virulence factor database [24]. Virulence-correlated gene (vcg) typing was performed via in silico PCR (https://www.bioinformatics.org/sms2/pcr_products.html) with previously reported primer sets [25, 26].

A core single-nucleotide polymorphism (SNP) alignment for 26 V*. vulnificus* genomes was performed using snippy (https://github.com/tseemann/snippy). A maximum likelihood phylogenetic tree based on core SNPs was constructed using RAxML with a generalized time-reversible gamma model [27]. The resulting phylogenetic tree was visualized using Microreact [28].

### Statistical analyses

Categorical variables were compared via Fisher’s exact test. All tests were two-tailed, and *P*-values < 0.05 were considered statistically significant. All statistical analyses were conducted using the SPSS software (IBM Corp., Armonk, NY, USA).

## Results

### Sample distribution

**Fig. 1:**
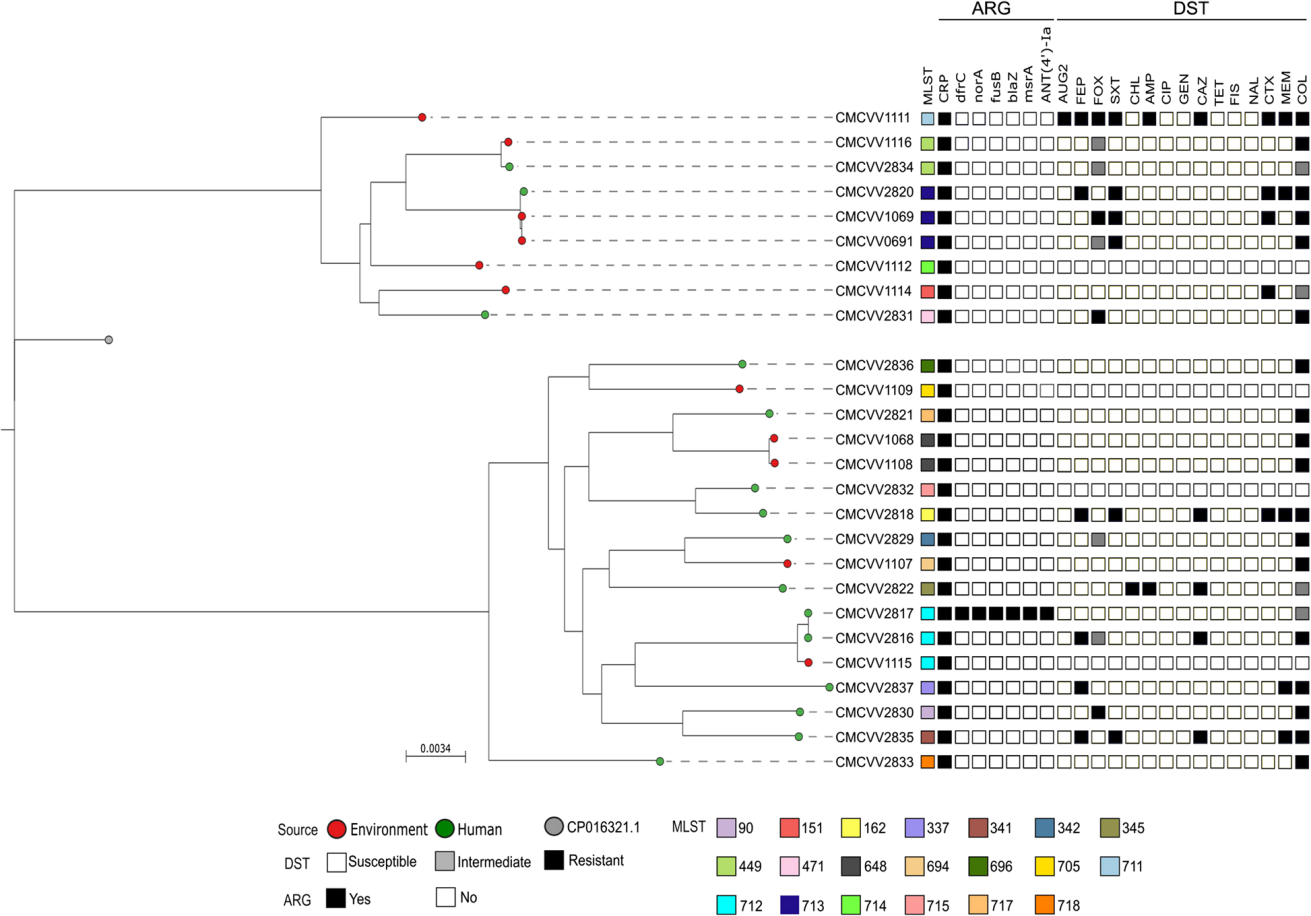
Maximum likelihood tree of 26 V*. vulnificus* strains collected from South Korea. Sample sources are represented by colored circles, while other phenotypic and genotypic characteristics are represented by colored squares. ARG, antimicrobial resistance gene; DST, drug susceptibility test

Of the 26 V*. vulnificus* strains isolated between 2010 and 2022, 11 environmental strains (42.3%) were collected from the coastal water of Gyeongsangnam-do, Jeollanam-do, Busan, and Ulsan, and 15 clinical strains (57.7%) were collected from Gyeongsangnam-do in South Korea. The phylogenetic tree showed relatively clear differences between the clinical and environmental strains (Fig. 1). Clinical strains exhibited a high degree of phylogenetic coherence, with 70% (11/15) forming a monophyletic clade, suggesting their conserved genetic lineage and potential for clonal expansion. In contrast, environmental strains were phylogenetically dispersed across multiple distinct branches, indicating high genetic heterogeneity and evolutionary divergence.

### Sequence types

WGS analysis revealed high-quality sequence reads of 26 V*. vulnificus* strains. Average N50 value was 375.8 kb (range: 149.0–770.0 kb), and average genome size was 5.17 Mb (range: 4.83–7.53 Mb). In total, 20 STs were identified, of which the following 8 STs were newly discovered using housekeeping genes: ST705 (*n* = 1), ST711 (*n* = 1), ST714 (*n* = 1), ST715 (*n* = 1), ST717 (*n* = 1), ST718 (*n* = 1), ST712 (*n* = 3), and ST713 (*n* = 3) (Supplementary Table S1). The identified new alleles and STs have been deposited into the PubMLST database for assignment. Only four STs were recurrently detected (ST449 [*n* = 2], ST648 [*n* = 2], ST712 [*n* = 3], and ST713 [*n* = 3]), whereas the other were singletons (Fig. 1). These results suggest that the genetic background of *V. vulnificus* is largely heterogeneous with various STs yet to be discovered.

### Antimicrobial resistance

Phenotypic drug susceptibility testing of the strains using 15 antibiotics revealed distinct resistance patterns of the clinical and environmental strains (Fig. 1 and Table 1). Clinical strains exhibited higher resistance to cefepime, ceftazidime, and meropenem than the environmental strains. Five clinical strains (35.7%, 5/14) and only one environmental strain (11.1%, 1/9) were resistant to cefepime. Similarly, 28.6% (4/14) of clinical strains and only one environmental strain (11.1%, 1/9) were resistant to ceftazidime and meropenem each. In contrast, resistance to trimethoprim/sulfamethoxazole (33.3%, 3/9) and cefotaxime (33.3%, 3/9) was higher in the environmental strains than in the clinical strains (21.4% and 14.3%, respectively) (Table 1). Notably, both the environmental (8/9, 88.9%) and clinical (11/14, 78.6%) strains showed high colistin resistance.

**Table 1:** Phenotypic drug susceptibility test for *V. vulnificus* strains

Antibiotic class	Drugs	Environment (*n* = 8)			Clinical (*n* = 14)		
		**S**	**I**	**R**	**S**	**I**	**R**
Fluoroquinolones	CIP	8 (100%)	0	0	14 (100%)	0	0
Cephalosporins	FOX	4 (50)%	2 (25%)	2 (25%)	9 (64.3%)	3 (21.4%)	2 (14.3%)
	FEP	7 (87.5%)	0	1 (12.5%)	9 (64.3%)	0	5 (35.7%)
Amphenicols	CHL	8 (100%)	0	0	13 (92.9%)	0	1 (7.1%)
Aminoglycosides	GEN	8 (100%)	0	0	14 (100%)	0	0
Tetracyclines	TET	8 (100%)	0	0	14 (100%)	0	0
Quinolones	NAL	8 (100%)	0	0	14 (100%)	0	0
Cephems	CAZ	7 (87.5%)	0	1 (12.5%)	10 (71.4%)	0	4 (28.6%)
	CTX	5 (62.5%)	0	3 (37.5%)	12 (85.7%)	0	2 (14.3%)
Carbapenem	MEM	7 (87.5%)	0	1 (12.5%)	10 (71.4%)	0	4 (28.6%)
Penicillin	AMP	7 (87.5%)	0	1 (12.5%)	13 (92.9%)	0	1 (7.1%)
	AMC	7 (87.5%)	0	1 (12.5%)	14 (100%)	0	0
Polymyxin	COL	0	1 (12.5%)	7 (87.5%)	0	3 (21.4%)	11 (78.6%)
Sulfonamide	FIS	8 (100%)	0	0	14 (100%)	0	0
	SXT	5 (62.5%)	0	3 (37.5%)	11 (78.6%)	0	3 (21.4%)

*CIP* ciprofloxacin, *FOX* cefoxitin, *FEP* cefepime, *CHL* chloramphenicol, *GEN* gentamicin, *TET* tetracycline, *NAL* nalidixic acid, *CAZ* ceftazidime, *CTX* cefotaxime, *MEM* meropenem, *AMP* ampicillin, *AMC* amoxicillin/clavulanic acid, *COL* colistin, *FIS* sulfisoxazole, *SXT* trimethoprim/sulfamethoxazole, *S* susceptible, *I* intermediate, *R* resistant

All strains harbored the efflux pump-related gene, cyclic AMP receptor protein (CRP). Additionally, *norA*, *ant(4′)-Ia*, *msrA*, *fusB*, *blaZ*, and *dfrC* were detected in CMCVV2817 (Supplementary Table S1). However, no associations were detected between the identified resistance genes and phenotypic resistance of the isolates.

### Multiple antibiotics resistance index

MAR index was calculated for the strains showing phenotypic resistance to at least two antibiotics. The most common resistance pattern found in 11 strains included cefoxitin, cefotaxime, colistin, cefepime, ceftazidime, meropenem, and trimethoprim/sulfamethoxazole, with an MAR index of 0.5 (Table 2). This pattern was more frequently observed in the clinical strains (46.7%, 7/15) than in the environmental strains (36.4%, 4/11). However, as the number of antibiotics in the resistance pattern decreased, the differences between the clinical and environmental strains also decreased. Of note, for cefoxitin and cefotaxime (MAR index of 0.1), resistance rate in the environmental strains (18.2%) was higher than in the clinical strains (13.3%) (Table 2). These results suggest that clinical strains tend to develop resistance to a broader range of antibiotics, whereas environmental strains may show similar or higher resistance rates when fewer antibiotics are involved.

**Table 2:** Multiple antibiotic resistance (MAR) index of *V. vulnificus* strains

Resistance pattern	Frequency of occurrence	Source	No. (%)	MAR index
Cefoxitin + cefotaxime + colistin + cefepime + ceftazidime + meropenem + trimethoprim/sulfamethoxazole	11	Environment	4/11 (36.4%)	0.5
		Human	7/15 (46.7%)	
Cefoxitin + cefotaxime + colistin + cefepime + ceftazidime + meropenem	9	Environment	3/11 (27.3%)	0.4
		Human	6/15 (40.0%)	
Cefoxitin + cefotaxime + colistin + cefepime + ceftazidime	8	Environment	3/11 (27.3%)	0.3
		Human	5/15 (33.3%)	
Cefoxitin + cefotaxime + colistin + cefepime	7	Environment	3/11 (27.3%)	0.3
		Human	4/15 (26.7%)	
Cefoxitin + cefotaxime + colistin	6	Environment	3/11 (27.3%)	0.2
		Human	3/15 (20.0%)	
Cefoxitin + cefotaxime	4	Environment	2/11 (18.2%)	0.1
		Human	2/15 (13.3%)	

### Virulence factors

To distinguish between the clinical (C-type) and environmental (E-type) *V. vulnificus* strains, we analyzed vcg and identified 19 vcg-C and 7 vcg-E types (Supplementary Table S1). Six vcg-C and 5 vcg-E types were identified in the environmental strains, whereas 13 vcg-C and 2 vcg-E types were identified in the clinical strains. High proportion of vcg-C types in environmental strains (54.5%, 6/11) indicated the presence of highly virulent strains in the environment. In contrast, low presence of vcg-E types in human isolates (13.3%, 2/15) indicated that the vcg type alone is not a definitive predictor of pathogenicity.

**Fig. 2:**
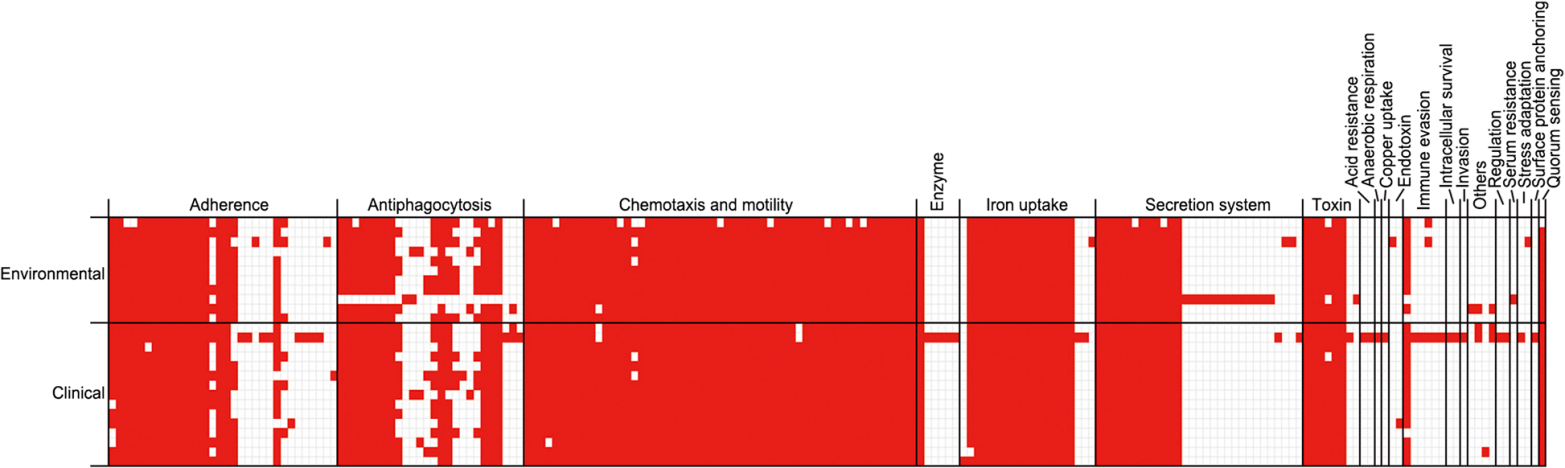
Heat map of 201 virulence genes present in 26 V*. vulnificus* isolates. The *X*-axis and *Y*-axis represent the virulence functional categories and sample sources, respectively

In total, we identified 201 virulence genes in 26 V*. vulnificus* strains, with an average of 125.6 (110–161) genes in each strain (Supplementary Table S1). Among these, 85 genes were ubiquitous, whereas 116 genes were detected at variable rates; 36, 2, 1, 4, and 73 genes were detected in 80–99, 60–80, 40–60, 20–40, and 0–20% of the isolates, respectively (Fig. 2). Overall, virulence gene counts per strain for each virulence functional class were similar between the clinical and environmental groups (124.3 vs. 126.5), indicating comparable virulence potential. Furthermore, clinical strains exhibited a similar number of virulence genes as the environmental strains in several key categories, including adherence (18.5 vs.18.4), antiphagocytosis (15.5 vs.14.4), and chemotaxis/motility (54.5 vs. 53.9), indicating similar host interactions and immune evasion capacities (Table 3). Notably, one clinical strain with a novel ST (CMCVV2817) exhibited a unique combination of virulence genes not detected in the environmental strains. These genes were related to acid resistance, anaerobic respiration, copper uptake, intracellular survival, invasion, and surface protein anchoring (Supplementary Table S1), suggesting their potential roles in host cell invasion and survival.

**Table 3:** Differences in the number of virulence factors between clinical and environmental isolates

Virulence class (number of related genes)	Environmental isolates (*n* = 11)		Clinical isolates (*n* = 15)	
	**Virulence genes**	**Per strain (%)**^a^	**Virulence genes**	**Per strain (%)**^a^
Acid resistance (*n* = 2)	0	0	2	0.13
Adherence (*n* = 51)	202	18.36	277	18.47
Anaerobic respiration (*n* = 1)	0	0	1	0.07
Antiphagocytosis (*n* = 32)	158	14.36	233	15.53
Chemotaxis and motility (*n* = 56)	593	53.91	818	54.53
Copper uptake (*n* = 1)	0	0	1	0.07
Endotoxin (*n* = 2)	1	0.09	1	0.07
Enzyme (*n* = 7)	11	1	20	1.33
Immune evasion (*n* = 6)	11	1	19	1.27
Intracellular survival (*n* = 2)	0	0	1	0.07
Invasion (*n* = 1)	0	0	1	0.07
Iron uptake (*n* = 25)	166	15.09	227	15.13
Quorum sensing (*n* = 2)	10	0.91	15	1
Regulation (*n* = 2)	0	0	2	0.13
Secretion system (*n* = 86)	145	13.18	182	12.13
Serum resistance (*n* = 1)	1	0.09	0	0
Stress adaptation (*n* = 2)	1	0.09	1	0.07
Surface protein anchoring (*n* = 1)	0	0	1	0.07
Toxin (*n* = 14)	65	5.91	90	6
Others (*n* = 4)	3	0.27	5	0.33

^a^Total VF genes divided by the number of isolates

## Discussion

Although *V. vulnificus* inhabits coastal environments worldwide, the research on this pathogen is scarce [29]. To the best of our knowledge, this study is the first to provide comprehensive insights into the genetic diversity, virulence factors, and antibiotic resistance profiles of *V. vulnificus* strains in South Korea. The identification of the eight novel STs expanded the ST profile, highlighting the genetic diversity of *V. vulnificus* in South Korea. Comparison of the molecular features of strains collected from various sample sources revealed the following key points: (1) clinical strains exhibit resistance to a broader range of antibiotics than the environmental strains; however, environmental strains show notably higher resistance to cefotaxime than the clinical strains, (2) wide diversity of STs and the presence of both vcg-C and vcg-E types in the environmental and clinical strains indicate that the vcg typing system alone cannot definitively predict pathogenicity, (3) no quantitative or qualitative differences are observed in the virulence factors of environmental and clinical strains, and (4) high ST diversity, including the novel STs detected in this study, complicates the understanding of the pathogenic potential of *V. vulnificus*.

Environmental and clinical strains of *Vibrio* spp. have similar virulence gene profiles, making it difficult to distinguish them based solely on the presence of virulence genes [30, 31], which is consistent with the results of this study. However, we identified unique virulence factors related to acid resistance, anaerobic respiration, intracellular survival, and invasion in a clinical strain with a novel ST (CMCVV2817), indicating its potential adaptations for survival in human hosts. These results suggest that it may have acquired specific genetic adaptations that enhance its pathogenicity. The accumulation of multiple virulence factors in a single strain indicates an increased risk of severe infection, making it a critical target for future monitoring and research.

Although a previous study reported the polymyxin class resistance of *V. vulnificus* [32], both clinical and environmental strains were non-susceptible to third-generation cephalosporins, including ceftazidime and cefotaxime, in this study. As the Centers for Disease Control and Prevention recommends the use of third-generation cephalosporins for the treatment of *V. vulnificus* wound infections [33], our finding highlights the potential challenges for the treatment of *V. vulnificus* infections. In this study, 11.1% (1/9) of environmental strains and 28.6% (4/14) of clinical strains were resistant to ceftazidime. Moreover, 33.3% (3/9) of environmental strains and 14.3% (2/14) of clinical strains were resistant to cefotaxime. MAR index ranged from 0.1 to 0.5, indicating varying degree of MAR among the strains. For strains with MAR index > 0.2, contamination from high-risk sources poses a health risk to humans [32]. Collectively, these findings support the need for stringent surveillance programs to track the emergence and spread of resistant *V. vulnificus* strains.

This study was limited by its small sample size, which may not fully represent the broad distribution range of *V. vulnificus* strains, warranting future studies in diverse geographic locations with large sample sizes. Moreover, longitudinal surveillance is essential to monitor the transmission of antibiotic-resistant strains, particularly those resistant to third-generation cephalosporins. Functional studies should investigate the unique virulence factors identified in certain clinical strains to clarify their roles in host–pathogen interactions and pathogenicity. Nevertheless, this study not only detected novel STs but also provided valuable insights into the broad spectrum of STs, virulence factors, and antibiotic resistance in *V. vulnificus*, enhancing our understanding of its pathogenic potential in clinical and environmental settings and informing future treatment and surveillance strategies.

## Supplementary Information

Additional file 1: Supplemental Table S1. Genomic characteristics of 26 vibrio vulnificus isolates.

## Data Availability

All datasets generated or analyzed in this study are included in the Supplementary Information. Raw FASTQ files are available upon request from the corresponding authors.
